# Risk factors for the development of tubo-ovarian abscesses in women with ovarian endometriosis: a retrospective matched case–control study

**DOI:** 10.1186/s12905-021-01188-6

**Published:** 2021-01-30

**Authors:** Yang Gao, Pengpeng Qu, Yang Zhou, Wei Ding

**Affiliations:** 1 Department of Gynecological Oncology, Tianjin Central Hospital of Gynecology Obstetrics, No. 156 Nankai San Ma Road, Nankai District, 300100 Tianjin, China; 2 Clinical College of Central Gynecology and Obstetrics, Tianjin Medical University, Tianjin, China; 3 Department of Intensive Care, People’s Hospital of Tianjin Affiliated to Nankai University, Tianjin, China

**Keywords:** Tubo-ovarian abscess, Ovarian endometriotic cyst, Risk factors, Infection, In vitro fertilization

## Abstract

**Background:**

The purpose of this study was to assess the risk factors associated with the development of tubo-ovarian abscesses in women with ovarian endometriosis cysts.

**Methods:**

This retrospective single-center study included 176 women: 44 with tubo-ovarian abscesses associated with ovarian endometriosis and 132 age-matched (1:3) patients with ovarian endometriosis but without tubo-ovarian abscesses. Diagnoses were made via surgical exploration and pathological examination. The potential risk factors of tubo-ovarian abscesses associated with ovarian endometriosis were evaluated using univariate analysis. The results (*p* ≤ 0.05) of these parameters were analyzed using a multivariate model.

**Results:**

Five factors were included in the multivariate conditional logistic regression model, including in vitro fertilization, presence of an intrauterine device, lower genital tract infection, spontaneous rupture of ovarian endometriosis cysts, and diabetes mellitus. The presence of a lower genital tract infection (odds ratio 5.462, 95% CI 1.772–16.839) and spontaneous rupture of ovarian endometriosis cysts (odds ratio 2.572, 95% CI 1.071–6.174) were found to be statistically significant risk factors for tubo-ovarian abscesses associated with ovarian endometriosis.

**Conclusions:**

Among the factors investigated, genital tract infections and spontaneous rupture of ovarian endometriosis cysts were found to be involved in the occurrence of tubo-ovarian abscesses associated with ovarian endometriosis. Our findings indicate that tubo-ovarian abscesses associated with ovarian endometriosis may not be linked to in vitro fertilization as previously thought.

## Background

Tubo-ovarian abscess (TOA) is a complex and severe complication found in 15–34% of patients with pelvic inflammatory disease (PID) [1, 2]. PID and TOA occur more frequently and are more severe in women with endometriosis than in those without endometriosis [3]. Researchers have demonstrated that no difference in tubal patency and morphological alterations between patients with ovarian endometriosis and deep infiltrating endometriosis (DIE). The above findings suggested that endometriosis can aggravate tubal adhesions and distortions through intrinsic pathological mechanisms such as inflammatory microenvironment independent of disease type or severity [4]. A TOA associated with ovarian endometriosis (OE-TOA) is a potentially life-threatening condition [5], which is also related to other morbidities, such as infertility, chronic pelvic pain, and ectopic pregnancy [6].

Several risk factors for PID and TOA have been identified, including young age, multiple sexual partners, sexually transmitted infections, chlamydia and gonorrhea infections, uterine instrumentation, interruption of the cervical barrier, hysterosalpingography, hysteroscopy, and in vitro fertilization (IVF) [7–9]. However, more comprehensive studies on the risk factors for OE-TOA are still needed. Only a few studies have reported that IVF or oocyte retrieval plays an important role in the development of OE-TOA [10, 11]. This is not surprising considering the high rate of infertility among individuals with endometriosis, with a prevalence rate of 30–50% [12, 13]. This may result in a vicious cycle of endometriosis leading to infertility, causing the need for IVF, which then leads to TOA, and further infertility. Unfortunately, whether IVF and oocyte retrieval are risk factors for OE-TOA still remains controversial.

The aim of this study was to explore the risk factors associated with OE-TOA and to provide an experimental basis for its early diagnosis, prevention, and cure. The secondary objective was to evaluate whether IVF increases the risk of OE-TOA.

## Methods

This was a retrospective comparative study performed in a single medical center. The study was approved by the hospital’s ethics committee, and informed consent was obtained from each patient that took part in this study.

**Fig. 1:**
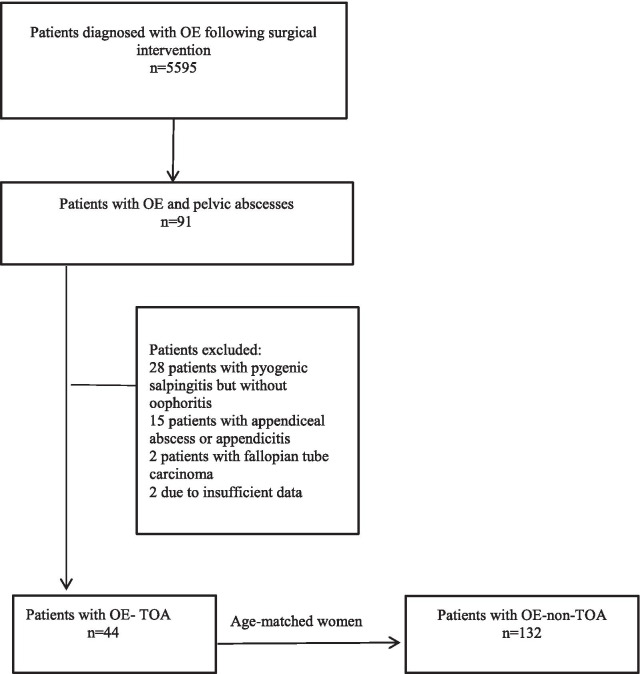
Study flowchart

The medical records of 5595 consecutive patients diagnosed with ovarian endometriosis (OE) who underwent laparoscopy or laparotomy at Tianjin Central Hospital of Gynecology and Obstetrics between January 1, 2010, and December 1, 2019, were retrospectively reviewed. Of these patients, 176 were evaluated in this study and were divided into a case group (composed of 44 patients with OE-TOA) and a control group (composed of 132 non-OE-TOA patients), based on the following inclusion criteria: the indication for surgery was the presence of an adnexal mass (greater than 4 cm in diameter). The case group and the control group were determined according to the following criteria. (1) The case group: pus observed during surgery and a confirmed diagnosis of OE-TOA by pathological examination (the pathological criteria included endometrial glands and stroma within the ovarian cyst, and neutrophils infiltrating into the capsule, with or without acute pyogenic salpingitis). (2) The control group: no pus observed during surgery and pathological examination indicated only OE cysts. The exclusion criteria were: (1) cancers of pelvic organs; (2) appendiceal abscesses; (3) appendicitis; and (4) cases with incomplete or unknown data (Fig. 1).

All diagnoses were confirmed during surgery and later by a pathologist. For each TOA case, three contemporaneous non-TOA control patients were selected from the electronic health record-derived data and matched by age (± 3 years).

For both cases and controls, clinical data, demographic data, and putative risk factors for OE-TOA were extracted from the electronic health record, including age, marital status, gravidity, parity, infertility, previous PID, history of ectopic pregnancy, previous removal of OE cysts, previous appendectomy, cesarean delivery, IVF, uterine cavity surgery within 15 days, presence of an intrauterine device (IUD), lower genital tract infection, spontaneous rupture of ovarian endometriotic cysts, dysmenorrhea, diabetes mellitus, hypertension, smoking status, and carbohydrate antigen 125 (CA125).

### Statistical analyses

Continuous variables that followed a normal distribution pattern and had homogenous variance were expressed as means ± standard deviations and were compared using Student’s t-test. Non-normally distributed data were expressed as medians and analyzed using the Mann–Whitney *U* test. Intergroup differences in categorical variables were compared using the chi-square test or Fisher’s exact test. In addition, a *p* value of ≤ 0.05 was used in the univariate analysis for inclusion of putative risk factors. Multivariate conditional logistic regression analysis was used to evaluate risk factors. Data processing and statistical analyses were completed using SPSS version 19.0 (IBM, Armonk, NY, USA). *p* values of < 0.05 were considered statistically significant.

## Results

A total of 176 women were evaluated in this study. Among these women, 44 were diagnosed with OE-TOA during the study period. The control group consisted of 132 non-OE-TOA patients. The demographic data of the two groups were comparable (Table 1).

**Table 1:** Demographic data of cases and controls

	Cases (n = 44)	Controls (n = 132)	*p *value
Age, years	39.61 ± 8.99	39.56 ± 8.92	0.972
Married, n (%)	36 (81.8%)	110 (83.3%)	0.817
Gravidity, n	1.52 ± 1.29	1.66 ± 1.31	0.549
Parity, n	0.80 ± 0.63	0.81 ± 0.67	0.895
Infertility, n (%)	12 (27.3%)	35 (26.5%)	0.922

The distribution of risk factors associated with OE-TOA in both the case and control groups was also tabulated (Table 2). Histories of PID and ectopic pregnancy were found in similar proportions of women in both groups. No statistically significant differences were found in operation history, including removal of OE cysts, appendectomy, and cesarean delivery, between the two groups. A higher proportion of women with TOA than without TOA had undergone IVF (6.8% vs. 0.8%, *p* = 0.049). Three patients in the case group and two patients in the control group had undergone uterine cavity operations within 15 days of admission; this difference was not statistically significant (*p* = 0.100). The number of women with IUDs in the case group was greater than that in the control group (*p* = 0.042). In the case group, 14 (31.8%) patients reported lower genital tract infections; in the control group, only 4 (3.0%) patients reported lower genital tract infections (*p* = 0.000). A greater number of patients had ruptured OE cysts in the case group than in the control group (9.1% vs. 1.5%, *p* = 0.016), as revealed by ultrasound results and surgical findings. The numbers of women with diabetes mellitus in the case and control groups were 5 (11.4%) and 3 (2.3%), respectively (*p* = 0.037). Dysmenorrhea was diagnosed in a similar proportion of patients in both groups. The differences in hypertension and smoking status between the two groups were not significant. There was also no significant difference in the level of CA125.

**Table 2:** Medical history and clinical characteristics of cases and controls

	Cases (n = 44)	Controls (n = 132)	*p *value
Previous pelvic inflammatory disease, n (%)	3 (6.8%)	2 (1.5%)	0.100
Ectopic pregnancy, n (%)	2 (4.5%)	1 (0.8%)	0.155
Previous removal of ovarian endometriosis cysts, n (%)	7 (15.9%)	9 (6.8%)	0.130
Previous appendectomy, n (%)	3 (6.8%)	4 (3.0%)	0.504
Caesarean, n (%)	14 (31.8%)	34 (25.8%)	0.434
In vitro fertilization, n (%)	3 (6.8%)	1 (0.8%)	0.049
Uterine cavity surgery within 15 days, n (%)	3 (6.8%)	2 (1.5%)	0.100
Intrauterine device, n (%)	14 (31.8%)	23 (17.4%)	0.042
Lower genital tract infection, n (%)	14 (31.8%)	4 (3.0%)	0.000
Spontaneous rupture of ovarian endometriosis cysts, n (%)	4 (9.1%)	2 (1.5%)	0.016
Dysmenorrhea, n (%)	19 (43.2%)	55 (41.7%)	0.860
Diabetes mellitus, n (%)	5 (11.4%)	3 (2.3%)	0.037
Hypertension, n (%)	3 (6.8%)	9 (6.8%)	1.000
Smoking, n (%)	3 (6.8%)	5 (3.8%)	0.676
Carbohydrate antigen 125, U/ml	60.35 ± 32.29	53.08 ± 38.98	0.224

Finally, multivariate conditional logistic regression analysis (Table 3) revealed that, among the putative risk factors evaluated, lower genital tract infection (odds ratio [OR] 5.462, 95% confidence interval [CI] 1.772–16.839), and rupture of ovarian endometriotic cysts (OR 2.572, 95% CI 1.071–6.174) were significantly associated with the development of OE-TOA. We found no relationship between IVF and OE-TOA (*p* = 0.130).

**Table 3:** Risk factors for the development of OE-TOA (multivariate conditional logistic regression)

	Odds ratio	95% Confidence interval	*p *value
In vitro fertilization	2.267	0.435–3.987	0.130
Intrauterine device	1.456	0.734–3.089	0.160
Lower genital tract infection	5.462	1.772–16.839	0.003
Spontaneous rupture of ovarian endometriosis cysts	2.572	1.071–6.174	0.035
Diabetes mellitus	1.548	0.876–4.469	0.194

OE-TOA: tubo-ovarian abscess associated with ovarian endometriosis

## Discussion

OE is a common benign gynecological disease, but a secondary TOA formation is seldom reported. Schmidt et al. reported the incidence of OE-TOA to be 2.15% in 1981 [14]; this was consistent with previous reports that indicated that the incidence of OE-TOA was 2.3% [15]. Of the 5,595 patients with OE in this study, 44 (0.79%) were diagnosed with OE-TOA. The incidence in this study was lower compared to that in previous reports. Although OE-TOA is rare, it is serious and sometimes fatal. This area of study requires our attention, as it has long been neglected.

Patients with OE are more susceptible than the general population to TOA [15]. Possible pathogeneses are as follows. (1) OE, which is itself is an immunodeficiency disease, leading to impairment in the ability of the immune system to wade off infections, at which point TOA easily emerges. (2) The OE capsule wall is thin and delicate, making it easy for bacteria to penetrate. (3) At the same time, OE blood content is an ideal culture medium that facilitates bacterial growth [16]. (4) The “bacterial contamination hypothesis” states that the incidence and occurrence of intrauterine microbial colonization and endometritis are significantly higher among women with endometriosis, especially after gonadotrophin-releasing hormone agonist treatment [17]. The result seem to be in contrast with those of Mohamed Mabrouk et al., who affirmed that preoperative hormone intervention can shrink the endometriosis lesion and reduce inflammation through ovarian inactivation. In addition, hormonal therapy can decrease the implantation of endometrial lesions, down-regulate cell proliferation, and increase the apoptosis of endometriosis tissues [18].

We observed a more than fivefold increase in OE-TOA risk after lower genital tract infection, which is in line with reports of previous studies. This may be because the cervical mucosal barrier is impaired during pathogenic microorganism infection; hence, infection can spread along the endometrium to other pelvic organs such as the fallopian tubes and ovaries [19]. This is a classic pattern of spread. According to related studies in the United States and Nordic countries, the pathogenic microorganisms of PID or TOA most commonly identified were *Neisseria gonorrhea* and *Chlamydia trachomatis* [20, 21]. This is not the case in China. Several domestic studies have indicated low detection rates for both microbes. A new study focusing on next-generation sequencing analysis of cervical mucus indicates that in a variable microbiota, two organisms, *Enterobacteriaceae* and *Streptococcus*, are more frequently detected in women with endometriosis [22]. Results of this study show that the microbial detection rate in the lower genital tract was significantly higher in cases than in controls. Furthermore, the most frequent pathogen was *Escherichia* *coli* (50%), followed by *Mycoplasma genitalium* (21.4%) and *Gardnerella vaginalis* (21.4%). This is partially in agreement with reports of previous studies, emphasizing the need to promptly investigate and effectively treat these infections with appropriate antibiotics.

Spontaneous rupture of ovarian endometriotic cysts was found to be a significant contributor to the risk of OE-TOA (OR = 2.572). To the best of our knowledge, rupturing of an ovarian endometriotic cyst as a risk factor for TOA has not been previously evaluated. Spontaneous rupture of an OE cyst is not usually a gynecological emergency. The incidence rate is seldom available in the literature from different countries, and the incidence rates reported in Chinese studies are inconsistent. The disease, characterized by abdominal pain and inflammation [23], is easily misdiagnosed due to its nonspecific clinical features and the lack of knowledge regarding biomarkers for early diagnosis [24]. Through analysis, our data shows that the incidence rates of OE cyst rupture in the case and control groups were 9.1% and 1.5%, respectively. Women with spontaneous rupture of ovarian endometriotic cysts had an increased risk of developing TOA. The exact underlying mechanism of this remains unclear. One possible explanation is that the capsule wall easily ruptures because of bleeding of the cyst and increased pressure during the pre-menstruation and menstruation phases of the menstrual cycle [25]. When the cyst bursts, a chocolate-like fluid pours into the abdominal cavity, leading to peritonitis. In addition, the blood content of an OE cyst is a good culture medium for mixed anaerobic bacteria, aerobic bacteria, and facultative bacterial infections [26]. If treatment is not initiated promptly, this could progress to a much more severe condition such as a TOA.

Pelvic abscesses often have tubal changes, however, few studies have been conducted on the relation of endometriosis and tubal alterations. Mohamed Mabrouk et al. evaluated tubal changes in 473 cases of endometriosis [4]. It was found that the change of fallopian tube had nothing to do with the degree of invasion of endometriosis, but was related to the operation history of endometriosis. This paper found that 15.9% of the patients in the case group had the operation history of endometriosis, while only 6.8% of the patients in the control group had the operation history, although there was no statistical difference. Therefore, larger sample data are needed in future investigations.

Finally, whether a patient with OE will develop TOA after IVF has been a controversial topic. Several studies propose that IVF and oocyte retrieval are major risk factors for the development of OE-TOA. Moreover, the condition is often more serious in these cases. The above conclusions are supported by the theory that the blood in an endometrioma offers a nutrient-rich culture for bacterial growth after transvaginal inoculation [27]. However, it is thought that the rate of TOA is low in patients suspected of having an ovarian endometriotic cyst after IVF and egg retrieval, even though no such patients have undergone laparoscopic exploration [28, 29]. Another view is that endometriosis-related infectious disease may be unrelated to assisted reproductive technology and that once OE-TOA occurs, the best form of intervention is early surgical drainage combined with intravenous antibiotics [30]. The results of our study directly contradict the view that patients with OE are more likely to have TOA after IVF or oocyte retrieval. These patients may benefit from comprehensive disinfection, antibiotic treatment, and ultrasound guidance to avoid intestinal puncture during oocyte retrieval.

The limitations of the study include a single-institutional retrospective design and a small mumber of patients.

## Conclusions

In conclusion, we found that IVF was not associated with an increased risk of OE-TOA. The risk factors significantly associated with OE-TOA were lower genital tract infections and spontaneous rupture of ovarian endometriotic cysts. To suppress the formation of OE-TOA and improve prognosis, suspected patients should be provided with prompt treatment, including prophylactic antibiotics (against *Escherichia* *coli*) as well as appropriate surgical interventions.

## Data Availability

The datasets generated and analyzed during the current study are available from the corresponding author on reasonable request.

## Abbreviations

IUD: Intrauterine device
IVF: In vitro fertilization
OE: Ovarian endometriosis
PID: Pelvic inflammatory disease
TOA: Tubo-ovarian abscess
